# Correction: Immunoassay-aptasensor for the determination of tumor-derived exosomes based on the combination of magnetic nanoparticles and hybridization chain reaction

**DOI:** 10.1039/d4ra90024c

**Published:** 2024-03-20

**Authors:** Hua Zhang, Yajuan Zhou, Dan Luo, Jingjian Liu, E. Yang, Guangyi Yang, Guangjun Feng, Qinhua Chen, Lun Wu

**Affiliations:** a Affiliated Dongfeng Hospital, Hubei University of Medicine Shiyan 442008 Hubei China wulun0909@163.com; b Department of Radiotherapy, Hubei Cancer Hospital, Tongji Medical College, Huazhong University of Science and Technology Wuhan 430074 China; c Shenzhen Baoan Authentic TCM Therapy Hospital Shenzhen Guangdong 518101 China cqh77@163.com +86-0719-8272238

## Abstract

Correction for ‘Immunoassay-aptasensor for the determination of tumor-derived exosomes based on the combination of magnetic nanoparticles and hybridization chain reaction’ by Hua Zhang *et al.*, *RSC Adv.*, 2021, **11**, 4983–4990, https://doi.org/10.1039/D0RA10159A.

The authors regret that an incorrect version of Fig. 4b was included in the original article. The correct version of Fig. 4b is presented below.
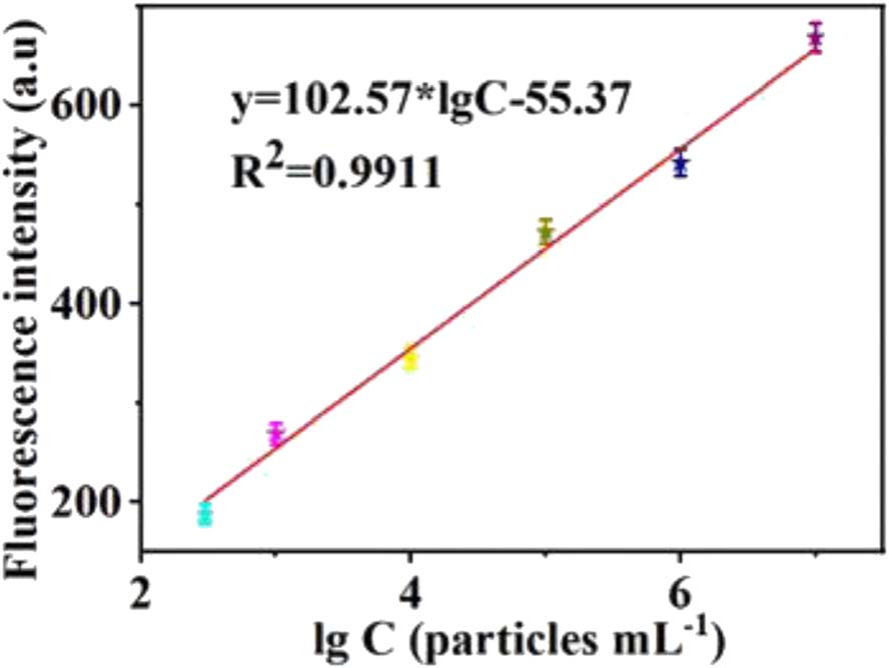


**Fig. 4b** The fluorescence intensity as a function of exosome concentration. It shows a strong correlation between the fluorescence intensity and the exosome concentration and the emission wavelength of 606 nm. Error bars: SD, *n* = 3.

Consequently, sections of the text in the manuscript should be adjusted according to this change, and these are detailed below.

The sentence on page 4988 beginning “The linear regression equation was *y* = 105.22 × lg *C* − 71.29 (*R*^2^ = 0.9963)…” should be corrected as “The linear regression equation was *y* = 102.57 × lg *C* − 55.37 (*R*^2^ = 0.9911), where *y* and lg *C*, respectively, represented the fluorescence intensity and the logarithm of exosome concentration”.

The Royal Society of Chemistry apologises for these errors and any consequent inconvenience to authors and readers.

## Supplementary Material

